# Supplementary material for: Neighborhood Beauty and the Brain in Older Japanese Adults
Source: Int J Environ Res Public Health. 2022 Dec 30;20(1):679. doi: 10.3390/ijerph20010679 (PMC9819975; doi:10.3390/ijerph20010679)
Supplement: Supplementary file 1 [file ijerph-20-00679-s001.zip › ijerph-2051273-SI.pdf]

Supplementary Table S1. Summary of brain region volumes among Japanese older adults (n = 476)

		Mean (SD)
mOFC (mm^3)	Left	4,883 (486)
	Right	4,922 (464)
Insula (mm^3)	Left	6,597 (539)
	Right	6,572 (609)

mOFC = medial orbitofrontal cortex; SD = standard deviation.

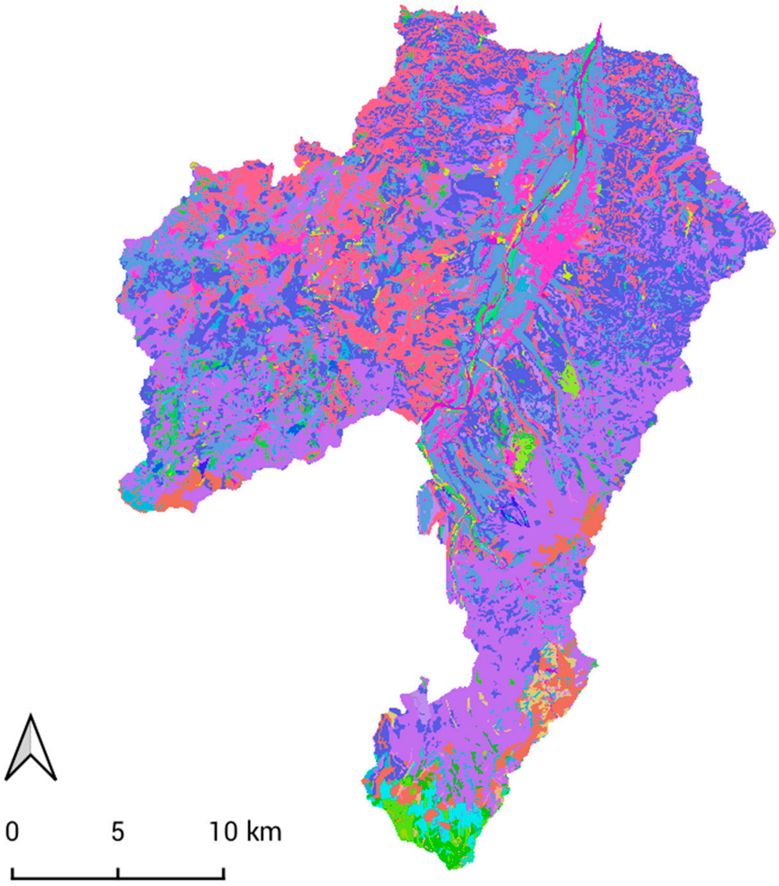

Supplementary Figure S1. Plant diversity in the study area (Tokamachi City). Colors represent differences in plant species. There are 52 plant species registered in Tokamachi City.

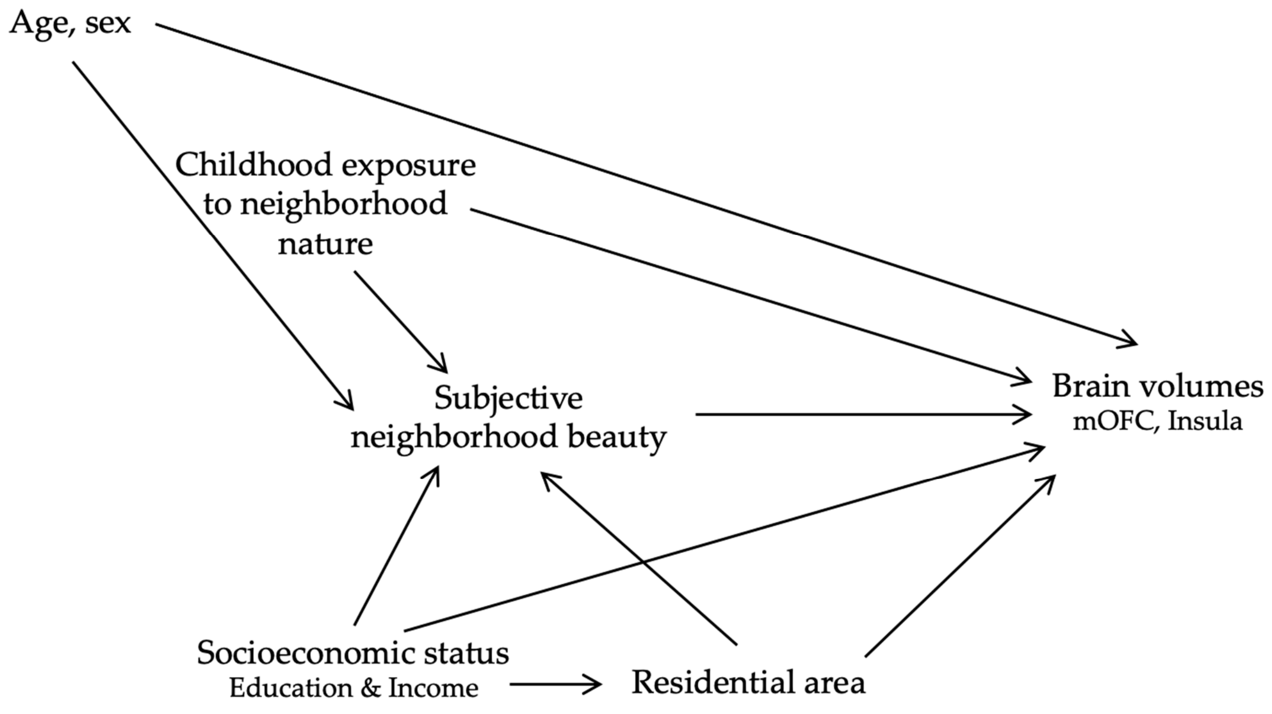

Supplementary Figure S2. Directed acyclic graph (DAG) showing the association between Subjective neighborhood beauty and brain volumes.

Supplementary Table S2. Associations between subjective neighborhood beauty and regional brain volume according to residential area among Japanese older adults (n = 476)

	mOFC		Insula	
	Left	Right	Left	Right
	Coef. (95% CI)	Coef. (95% CI)	Coef. (95% CI)	Coef. (95% CI)
Matsunoyama (mountain) (n = 174)				
Subjective beauty of neighborhood environment				
Low	referent	referent	referent	referent
Moderately low	**596 (280 to 912)**	**305 (13.6 to 597)**	51 (−324 to 427)	278 (−143 to 700)
Moderately high	**674 (408 to 940)**	**444 (198 to 690)**	**380 (64 to 696)**	**416 (61 to 771)**
High	**498 (274 to 721)**	**383 (177 to 589)**	**356 (91 to 621)**	**399 (101 to 696)**
p for trend	0.003	0.002	0.004	0.02
Central Tokamachi (downtown) (n = 302)				
Subjective beauty of neighborhood environment				
Low	referent	referent	referent	referent
Moderately low	98 (−62 to 257)	63 (−95.6 to 222)	123 (−51 to 297)	139 (−57 to 335)
Moderately high	63 (−101 to 227)	30 (−133.3 to 194)	163 (−17 to 342)	190 (−12 to 392)
High	153 (−2 to 308)	79 (−76 to 233)	145 (−24 to 315)	**296 (105 to 487)**
p for trend	0.09	0.42	0.11	0.002

Coef.: Regression coefficients; CI = confidence interval; mOFC = medial orbitofrontal cortex.

Boldface indicates statistical significance ($p < 0.05$).
